# Microbiome in Immune-Mediated Uveitis

**DOI:** 10.3390/ijms23137020

**Published:** 2022-06-24

**Authors:** Carmen Antía Rodríguez-Fernández, Manuel Busto Iglesias, Begoña de Domingo, Kelly Conde-Pérez, Juan A. Vallejo, Lorena Rodríguez-Martínez, Miguel González-Barcia, Victor Llorenç, Cristina Mondelo-Garcia, Margarita Poza, Anxo Fernández-Ferreiro

**Affiliations:** 1 Ophthalmology Department, University Clinical Hospital of Vigo (SERGAS), 36213 Vigo, Spain; carmenantia@gmail.com; 2 Pharmacy Department, University Clinical Hospital of Santiago de Compostela (SERGAS), 15706 Santiago de Compostela, Spain; manuelbustoiglesias@gmail.com (M.B.I.); miguel.gonzalez.barcia@sergas.es (M.G.-B.); crismondelo1@gmail.com (C.M.-G.); 3 Pharmacology Group, Health Research Institute of Santiago de Compostela (FIDIS), 15706 Santiago de Compostela, Spain; lorenamarinoalvarez@gmail.com; 4 Ophthalmology Department, University Clinical Hospital of Santiago Compostela (SERGAS), 15706 Santiago de Compostela, Spain; bdedbar@yahoo.es; 5 Microbiology Research Group: meiGAbiome, Biomedical Research Institute (INIBIC), Center for Advanced Research (CICA), University of A Coruña (UDC), CIBER of Infectious Diseases (CIBERINF), 15006 A Coruña, Spain; kelly.conde.perez@sergas.es (K.C.-P.); juan.andres.vallejo.vidal@sergas.es (J.A.V.); 6 Clínic Institute of Ophthalmology (ICOF), Clinic Hospital of Barcelona, 08028 Barcelona, Spain; llorens.victor@gmail.com; 7 Biomedical Research Institute August Pi i Sunyer (IDIBAPS), Clínic Hospital of Barcelona, 08036 Barcelona, Spain

**Keywords:** non-infectious uveitis (NIU), immune-mediated disease, gut microbiota, intestinal microbiome, ocular microbiome, microbiota modulation

## Abstract

In the last decades, personalized medicine has been increasing its presence in different fields of medicine, including ophthalmology. A new factor that can help us direct medicine towards the challenge of personalized treatments is the microbiome. The gut microbiome plays an important role in controlling immune response, and dysbiosis has been associated with immune-mediated diseases such as non-infectious uveitis (NIU). In this review, we gather the published evidence, both in the pre-clinical and clinical studies, that support the possible role of intestinal dysbiosis in the pathogenesis of NIU, as well as the modulation of the gut microbiota as a new possible therapeutic target. We describe the different mechanisms that have been proposed to involve dysbiosis in the causality of NIU, as well as the potential pharmacological tools that could be used to modify the microbiome (dietary supplementation, antibiotics, fecal microbiota transplantation, immunomodulators, or biologic drugs) and, consequently, in the control of the NIU. Furthermore, there is increasing scientific evidence suggesting that the treatment with anti-TNF not only restores the composition of the gut microbiota but also that the study of the composition of the gut microbiome will help predict the response of each patient to anti-TNF treatment.

## 1. Introduction

Uveitis encompasses a heterogeneous group of intraocular inflammatory diseases consisting of inflammation of the uveal tract. This can affect adjacent structures such as the retina or optic nerve. They are classified according to their etiology into infectious and non-infectious, with the latter being related, in most cases, to immune-mediated diseases. The origin of this inflammation can be attributed to an endogenous mechanism, either as part of a systemic disease (sarcoidosis, Ankylosing spondylitis (AS), Behçet Disease (BD), multiple sclerosis (MS), Vogt-Koyanagi-Harada syndrome (VKH), etc.) or in isolated ocular (such as Birdshot disease) [1]. The exact pathogenic mechanism of immune-mediated uveitis is not yet known; although an imbalance has been observed between autoreactive effector T cells (Th1 and Th17) involved in effector and pathogenic functions and regulatory T cells (Treg), involved in immunomodulatory functions (tolerance) [2,3]. Pro-inflammatory cytokines play a fundamental role in all these pathophysiological mechanisms. Elevated levels of interleukin-6 (IL-6), IL-17, IL-23, or tumor necrosis factor-alpha (TNF α) have been detected at higher concentrations in the blood and/or ocular fluids of patients with uveitis of various etiologies; and most therapeutic targets in recent decades have involved these cytokine pathways [4]. Chronic or recurrent inflammation and tissue damage may result from an exaggerated host immune response, and the microbiome may be a significant source of antigens and antigen-specific T cells.

The intestinal microbiota is the most abundant in humans and is composed of approximately 10^14^ microorganisms whose genomes constitute 100 times the size of the human genome [5]. The most frequent phyla, both in adult humans and in mice, are *Firmicutes* and *Bacteroidetes*, followed by *Actinobacteria* and *Proteobacteria*, while the least represented are the *Fusobacteria* and *Verrucomicrobia* [6]. Its composition is established at birth by maternal transmission and constantly changes to maintain homeostatic balance with the host’s immune system [7]. It is possible that diet is the environmental factor with the greatest influence [8], although it is also affected by chronic stress, circadian rhythm, exposure to medications, toxins, colonization by other external microorganisms, and different diseases [9,10]. The intestinal microbiota plays an important role in many physiological functions, and its function as a regulator of the immune system, modulating innate and adaptive responses have gained importance in recent years [11]. The alteration of the intestinal microbiota compared to what we consider a healthy and diverse microbiota is known as dysbiosis [12]. This may alter the homeostatic immune state and induce diseases [9].

Chronic or recurrent uveitis can be caused by local reactivations of persistent microbial agents or inadequately cleared antigens, which may intermittently break the proportion of T cells. Experimental data have demonstrated the crucial role of the gut microbiome in controlling both the innate and adaptive immune response, linking dysbiosis to immune-mediated diseases [13]. Some bacterial strains such as segmented filamentous bacteria promote the differentiation of Th17 in the gut. They have been associated with immune-mediated diseases such as non-infectious uveitis (NIU). On the contrary, other bacterial species such as some types of *Clostridia* and the species *Bacteroides Fragilis* are usually part of the commensal microbiota, promote the differentiation of Treg, and contribute to immune homeostasis [4]. Most current theories about the relationship between the microbiota and the immune system in the pathogenesis of the NIU are extraocular in nature, although recent studies suggest the presence of a previously unrecognized intraocular microbiome, which opens a new research path [14]. In recent years, based on experimental studies with animal and clinical models, a relationship between the intestinal microbiota and uveitis has become gradually established [1]. This relationship may predispose or even be the origin of uveitogenic or adjuvant pathogens [15]. In addition, the fact that all immunosuppressive drugs have been shown to have intrinsic antimicrobial activity, together with the effect of immunosuppression on the intestinal microbiota, and inflammatory diseases, supports this relationship between the intestinal microbiota and the NIU [16]. The identification of the causal or protective species would allow us to initiate new lines of research focused on the modification of the microbiota as a therapeutic possibility in immune-mediated uveitis.

This review gathers published evidence on the association between microbiota and uveitis, both in the pre-clinical and clinical fields. In the publications, the different mechanisms by which intestinal dysbiosis could participate in the pathogenesis of the NIU are described, as well as the potential treatments aimed at modulating the intestinal microbiota that could be used and/or interfere positively or negatively in its treatment.

## 2. Mechanisms of Microbial Pathogenesis and Uveitis

Growing clinical and pre-clinical evidence points to the microbiome and dysbiosis in the immune response and susceptibility to systemic diseases, including both those of gastrointestinal origin such as Inflammatory bowel disease (IBD), and those of another origin [17]. It has also been implicated in the neurodevelopment and function of the central nervous system (CNS), through a bidirectional connection known as the “gut-brain axis” whose communication is regulated by the microbiota [18]; thus associating it with mental health (anxiety, depression), neurological diseases (Alzheimer’s disease, Parkinson’s disease…) and aging [18,19,20]. More and more studies are now focusing their attention towards understanding the relationship between the microbiota and immune-mediated diseases that occur with uveitis, such as Behçet’s disease (BD) [21,22], AS [23], or Vogt-Koyanagi-Harada syndrome [24]. The decrease in the diversity of the microbiome has been linked in the literature to multiple diseases, such as IBD and AS, which are present from pediatric stages of life, suggesting that this dysbiosis may be associated with a genetic predisposition and is not only an effect, but also contributes to the pathogenesis of the disease [23,25].

NIUs target the neuroretina, considered a part of the CNS. In addition to NIUs, multiple eye conditions have recently been linked to intestinal dysbiosis, such as diabetic retinopathy (DR), age-related macular degeneration (AMD), and glaucoma [26,27,28,29]. Many studies have shown the potential role of micronutrient supplementation in slowing down AMD progression, as well as animal models proved systemic inflammation induced by diet and dysbiosis, contributing to AMD development [28]. This suggests the existence of a “gut-retinal axis”, yet to know if as a part of the “gut-brain axis” or as an independent bidirectional communication [1].

**Figure 1:**
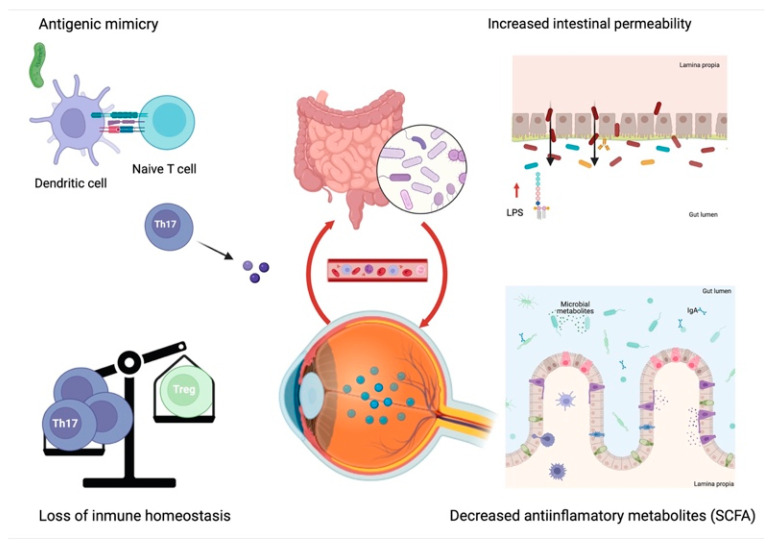
Mechanisms by which dysbiosis can participate in the pathogenesis of uveitis. Created with BioRender.com.

Interest in the study of the role of the microbiota in the development of NIU has been increasing in recent years, with several recent publications appearing that support its causality [15]. Dysbiosis participates in the pathogenesis of uveitis through four non-mutually exclusive mechanisms [30]: antigenic or molecular mimicry, the destruction of the intestinal barrier due to increased intestinal permeability, the loss of immune intestinal homeostasis, and the reduction of the production of beneficial anti-inflammatory metabolites (Figure 1).

### 2.1. Antigenic or Molecular Mimicry 

Antigenic mimicry is an important autoimmunity mechanism in which autoreactive T cells are generated by cross-reactivity of microbial peptides with autoantigens [31]. The pathogenesis of diseases associated with human leukocyte antigen-B27 (HLA-B27), including uveitis, has thus been related to a wide variety of peptides derived from microorganisms that show a high affinity for this molecule [32], including the species *Chlamydia trachomatis* and *Campylobacter jejuni* and the genera *Klebsiella*, *Salmonella*, *Yersinia* and *Shigella* [33,34]. Experimental studies in mice with spontaneous experimental autoimmune uveitis (EAU) have demonstrated this pathogenic mechanism [35]. Elimination of the microbial community by administration of broad-spectrum oral antibiotics attenuates the severity of uveitis and reduces intestinal Th17 activation, while the transfer of T cells from a transgenic mouse grown with microbiota induces uveitis in wildtype mice [35].

### 2.2. Increased Intestinal Permeability 

Inflammation of the mucosa caused by intestinal dysbiosis destroys the intestinal barrier, causing an increase in permeability and favors the translocation of microbiota or its products to the blood, lymphatic, submucosal, and lamina propria circulations. These products, such as lipopolysaccharides (LPS) and β-glucan, would reach different tissues through the vascular system, directly causing inflammation in target organs, and reaching the uvea and synovial tissue [32,36,37]. Experimental studies in mice with EAU demonstrated the relationship between the increase in intestinal permeability with uveitis. Janowitz et al. [38] studied intestinal changes in mice with EAU by immunization with inter-photoreceptor retinoid-binding protein (IRBP)_161–180_ peptide plus killed Mycobacterium tuberculosis (MTB) antigen as an adjuvant, whereas mice immunized with MTB alone or in combination with an irrelevant IRBP peptide that did not develop ocular inflammation were used as controls. An increased intestinal permeability was observed in IRBP-immunized mice through assessment of ZO-1 expression and a FITC-dextran assay. Both IRBP and MTB mice exhibited increased permeability compared to non-immunized mice, an increase that was more evident in IRBP-immunized mice. In contrast, only IRBP-immunized mice had increased permeability in the FITC-dextran assay that paralleled the course of uveitis. In addition, the increase in intestinal permeability coincided with changes in intestinal microbiota. Linear discriminant analysis of Effect Size (LEfSe) revealed an increased abundance in *Clostridium and S24-7* bacteria, but depletion in *Verrucomicrobia, Akkermansia, Dorea,* and other bacteria in uveitic mice compared to MTB control mice. Interestingly, these differences were more marked at the peak of uveitis, with increased *Prevotella, Lactobacilli, Anaeroplasma, Parabacteroides,* and *Clostridium* species in IRB-immunized mice, while *Ruminococcus, Bacteroidia, S24-7, Proteobacteria*, and *Desulfovibrio* were more abundant in MTB control mice. Furthermore, the degree of intestinal inflammation correlated with the severity of uveitis [38]. Therefore, intestinal permeability alteration may start at the onset of uveitis and becomes more intense with the progression of ocular inflammation accompanied by preceding dysbiosis to the outbreak of ocular inflammation [38].

### 2.3. Loss of Intestinal Immune Homeostasis

As previously described, T cells play an important role in the pathogenesis of autoimmunity. In conditions of dysbiosis there is a loss of intestinal homeostasis, which breaks the balance between Th17 and Treg, and leads to immune activation by increasing Th17 (and IL-17) and decreasing Treg (and IL-10); thus, causing inflammation [39,40]. This theory has been supported by studies in mice with induced EAU, showing that the administration of broad-spectrum oral antibiotics modifies the composition of the microbiota by reducing *Firmicutes* and *Bacteroidetes* phyla and *Alphaproteobacteria* class, and increasing *Gammaproteobacteria* class, and reducing the severity of uveitis by increasing the proportion of Treg in various lymphoid tissues and the eye [41]. In addition, the existence of migration of immune cells from the intestine to the eye has been proposed, since T cells of intestinal origin have been detected in the eye in mouse models with EAU [42].

### 2.4. Reduction of Anti-Inflammatory Microbial Metabolites

It is estimated that gut microbes produce thousands of metabolites that can regulate immune responses. The most common metabolites are short-chain fatty acids (SCFAs): butyrate, propionate, and acetate. These are metabolites obtained by the colonic microbiota through the fermentation of dietary fiber. These beneficial and protective metabolites have previously been studied in other inflammatory autoimmune diseases, such as HLA-B27-associated spondyloarthropathy [43]. This theory has also been demonstrated in experimental studies with mice with EAU in which the severity of uveitis decreased with exogenous supplementation of SCFAs [42,44]. These can reduce inflammation by inducing and increasing Treg cells in the intestinal lamina propria and lymph nodes, as well as suppressing effector T cells and decreasing their transport between the intestine and spleen [42].

## 3. Microbial Dysbiosis and Uveitis

Recent advances have made it possible to sequence the microbiome present in the human cornea and conjunctiva [45]. These have a different composition and a lower density of microorganisms than in the intestinal microbiome, but also with potential inflammatory regulation functions. An association between the ocular surface microbiome and the tear proteome has also been demonstrated [46]. The composition of the flora of the ocular surface differs according to the collection and research methods used, both due to the differences between the microbiome and the microbiota, and because of the difficulties of metagenomic sequencing (small sample size, contamination due to difficulty of the technique, etc.). In addition, the microbiome of the normal ocular surface varies with age, sex, environment, and diet, and can be altered by the use of therapies on the ocular surface (such as topical drugs or excessive use of contact lenses) [47], by chronic diseases such as diabetes or BD [48], and by systemic treatments such as the use of oral antibiotics [49]. Despite the difficulty in establishing a reference state, the literature agrees that the predominant genera on the healthy ocular surface are *Corynebacterium*, *Propionibacterium,* and *Staphylococcus* [50]. The mucosa of the ocular surface, being in direct contact with the environment, serves as a defense against the colonization of potentially pathogenic microbial species. The ocular microbiome influences ocular homeostasis, and it has been shown that the alteration of normal commensal flora induced by pathological states or antibiotics can cause an imbalance in favor of pathogenic species, increasing the risk of infections or ocular neoplasms [51].

The inside of the eye contains multiple immune system cells and inflammatory mediators, but the immune privilege of the eyeball prevents intraocular inflammation through mechanisms of immune ignorance and immune tolerance [52]. Despite the sterility “per se” of the interior of the anterior chamber, recent studies suggest the presence of a still unknown microbiome [14] that could modulate intraocular inflammatory responses. Nonetheless, this is for now still controversial. The aqueous humor microbiome has not been examined in patients with recurrent acute anterior uveitis (AAU). The study of the presence of microorganisms in aqueous humor during acute attacks of AAU could reveal important information related to the immunological and microbial mechanisms underlying this inflammatory disease [33].

Changes in the composition of the microbiota of the ocular surface have been found in pathologies such as blepharitis, trachoma, and dry eye [47]. In more detail, patients with blepharitis exhibited an increased abundance in *Staphylococcus*, *Streptophyta*, *Corynebacterium*, *Enhydrobacter*, and a decrease of *Propionibacterium*; patients with trachoma presented lower bacterial diversity and increased *Corynebacterium* and *Streptococcus*, while patients with dry eye syndrome had increased *Staphylococcus aureus*, *Corynebacterium*, *Propionibacterium*, *Rhodococcus* and *Klebsiella oxytoca*, the two latter considered as potential pathogens [47]. Furthermore, a relationship between ocular and non-ocular microbiome in retinal diseases, such as AMD, DR, or glaucoma, as well as uveitis has been suggested. Neither uveitis nor the rest of the subsequent diseases of the eye have been related to the ocular microbiome to date; due to the lack of studies [53]. However, there are several studies that show the relationship between the intestinal microbiota and NIUs both clinically and pre-clinically:

### 3.1. Pre-Clinical Studies

The involvement of the microbiota in the development of uveitis is based, fundamentally, on the EAU model induced by active immunization with external retinal antigen “inter-photoreceptor retinoid-binding protein” (IRBP) [41,54] and the spontaneous EAU model of a transgenic mouse that expresses the self-reactive T cell receptor (TCR) of [35,55]. Pre-clinical studies in which the causality of uveitis is related to the microbiota, as well as different therapeutic strategies for its modulation, are described in Table 1.

The absence of microbiota in “germ-free” mice or the decrease in bacterial load prior to disease induction by administration of oral antibiotics has been shown to significantly attenuate the susceptibility of developing EAU induced by IRBP [54].

In germ-free mice, lower retinal infiltration of T cells, lower levels of IFN-γ- and IL-17-producing T cells, and higher levels of regulatory T cells were found in the drainage lymph nodes of the eye [54]. This suggests that the microbiota regulates the inflammatory response by the adaptive pathway during autoantigen recognition. Nevertheless, this was not demonstrated in mice treated with antibiotics, suggesting that the reduction of the microbiota was incomplete in this group.

Nakamura et al. also observed a decrease in the severity of induced EAU following administration of broad-spectrum oral antibiotics, especially with vancomycin and metronidazole, but not with other antibiotics, which not only modified the composition of the microbiota but also produced an increase in the proportion of Treg in the intestinal lamina propria at the first week, and subsequently in extraintestinal lymphoid tissues and the eye [41].

On the other hand, Horai et al. tried to explain with a spontaneous EAU model [35] how immunologically privileged organs such as the eye are targets of autoimmunity, after a possible peripheral activation of autoreactive T cells that identify commensal microbiota. They found a decrease in disease severity in “germ-free” mice and when administering broad-spectrum oral antibiotics in combination, they observed an associated decrease in IRBP-specific T cells in the lamina propria of the intestine [55].

Experimental models also demonstrated that exogenous supplementation of SCFAs decreased the severity of uveitis [42,44]. Supplementation with sodium butyrate (NaB) attenuated the severity of EAU in mice, modified the balance of T cells, and switched from pathogenic Th17 to Treg [44]. These authors suggest that NaB reverses the differentiation from Th17 to Treg as previously demonstrated in vitro and in vivo [58], attenuating the severity of EAU via the Nrf2/HO-1 pathway.

Finally, experimental studies with transgenic rats for the HLA B27 gene have suggested the function of this in modulating the microbiome. The expression of HLA B27 was related to differences in the composition of the intestinal microbiome, with an increase in the relative abundance of *Prevotella* spp. and a decrease in *Rikenellaceae*, compared to wild rats, as well as an increase in *Bacteroides vulgatus* [59].

### 3.2. Clinical Studies

Clinical studies confirm some of the conclusions derived from pre-clinical studies. They have found a decrease in the abundance and diversity of bacterial [60] and fungal [61] microbiota in patients with undifferentiated and immune-mediated uveitis. There is also a decrease in anti-inflammatory or anti-pathogenic bacterial and fungal species, as well as an increase in pro-inflammatory and opportunistic species [60,61]. The clinical studies in which the causality of the NIU is related to the microbiota, as well as different therapeutic strategies for its modulation, are described in Table 2.

Despite finding no differences in the gut microbiome of patients with AAU compared to healthy subjects, Huang et al. found a different metabolic phenotype in AAU patients with increased expression of seven fecal metabolites (Table 2) by using gas chromatographic mass spectrometry-based metabolomics [62]. Clinical studies in patients with BD have shown an alteration in the composition of their microbiota [21,64], as well as significant differences in the composition of the gut microbiome between patients with BD without uveitis and those who develop uveitis (Table 2) [21]. This suggests an association of intestinal dysbiosis with the pathophysiology of this disease. Several authors have reported a decrease in butyrate-producing bacteria [22,63] and, consequently, a decrease in butyrate (anti-inflammatory), which could justify an increase in the inflammatory state. It has also been proposed that the gut microbiota can be modified by dietary patterns in these patients [66]. In patients with active VKH, a decrease in butyrate, lactate, and methane-producing bacteria was also seen, associated with an increase in Gram-negative bacteria, such as *Paraprevotella* spp. In addition, these differences decreased after immunosuppressive treatment, with an identifiable prognostic response to treatment markers [65].

The intestine has also been related to the pathogenesis of spondylarthritis: an alteration in the composition of the microbiota of patients with AS with respect to healthy controls, correlated with the state of their disease has been described [67]. An association has also been found between their intestinal inflammatory status and microbiota profile, with greater microbial richness in inflamed compared to non-inflamed tissues and higher in chronically than in acutely inflamed samples. Also, the composition of the bacterial community in inflamed samples differed from that of non-inflamed samples in AS patients, with no differences between chronic and acute inflammatory status. Unfortunately, information on the specific bacteria is not available from this study [66]. An increase in the bacterial genus *Dialister* has also been reported, which is positively correlated with inflammatory activity, suggesting that this bacterial species may serve as a marker of activity in AS [68].

## 4. Therapeutic Approaches Aimed at Modifying the Intestinal Microbiota

**Figure 2:**
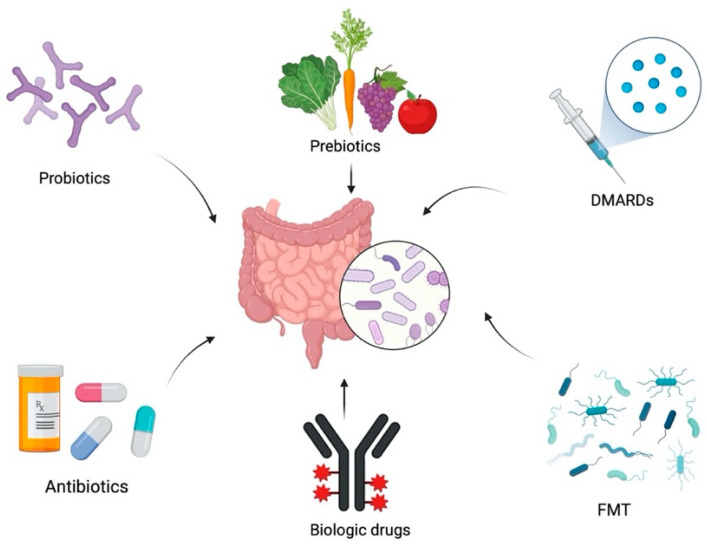
Therapeutic approaches that modify the intestinal microbiota. Created with BioRender.com. FMT: Faecal microbiota transplantation. DMARDs: Disease-modifying anti-rheumatic drugs.

Experimental models, as well as results in clinical studies, lead to the conclusion that the gut microbiota can modulate the responses and behavior of uveitogenic T cells at various levels, providing adaptive and innate stimuli, as well as possible regulatory effects. This connection not only allows us to advance in the knowledge of the pathogenesis of the disease, but also opens the way to possible new therapeutic targets focused on the modification of the microbiome. The pharmacological tools currently used to modify the microbiome (Figure 2), and that have shown their potential usefulness in the treatment of uveitis, are described below, either in EAU experimental models or in clinical studies, which are described in Table 1 and Table 2.

### 4.1. Probiotics

Probiotics introduce functional microbial components that are beneficial to the correct functioning of the gut and could be treatment options due to provoking an attenuation of the immune response. Bifidobacterium preserves intestinal barrier functions and produces SCFAs, and there is growing evidence demonstrating the beneficial effects of their supplementation on health, from protection against infection to different positive extra and intra-intestinal effects [69]. Their use in autoimmune diseases has been suggested since oral administration of probiotics in experimental murine models has been shown to have immunoregulatory functions [70]. Treatment with probiotics such as *Lactobacillus* spp. and *Bifidobacterium bifidum* produce induction of Treg in the intestinal mucosa and a decrease in inflammatory activity [70].

Supplementation with IRT-5 probiotics (*Lactobacillus casei, Lactobacillus acidophilus, Lactobacillus reuteri, Bifidobacterium bifidum,* and *Streptococcus thermophilus*) following removal of the microbiota with antibiotics reduced the severity of induced EAU in mice [57]. Likewise, treatment with the live probiotic *Escherichia coli Nissle* 1917 (EcN) prior to the induction of EAU in mice also protected against the development of uveitis and produced reinforcement of the integrity of the intestinal mucosa towards an anti-inflammatory state [56].

### 4.2. Prebiotics

Every day more articles show the impact of diet on health, possibly by modulating the microbiome and its metabolites. Prebiotics stimulate the proliferation of beneficial microorganisms and are present in foods of plant origin rich in fiber. Diets rich in unrefined cereals, fruits, vegetables, and legumes improve the profile of the intestinal microbiota since they are high in fiber, whose fermentation gives rise to the aforementioned SCFAs and other beneficial microbial metabolites with the ability to restore immune homeostasis [71,72,73]. Exogenous supplements of propionate [42,43] and butyrate [44] have been shown to modulate the immune system by attenuating the severity of EAU in animal models, as previously mentioned, turning them into potential treatment strategies.

Studies in patients with BD show a reduction in butyrate production that is linked to an increase in inflammatory status [22,63]. In this pathology, the change in the microbiota associated with three different diets is being analyzed, and one of them is supplemented with butyrate [66].

### 4.3. Antibiotic Therapy

Through the use of antibiotics, the microbiome can also be modulated. Experimental models in mice with EAU—both induced and spontaneous—have shown that oral administration of antibiotics attenuates ocular inflammation by modifying the composition of their gut microbiota. Different antibiotics have been used in isolation and combination in these studies: metronidazole and ciprofloxacin [54], ampicillin, metronidazole, neomycin, and vancomycin [35,41,55].

Trying to find the causative bacteria of the disease, Zárate-Baldés et al. perform their study with ampicillin, metronidazole, neomycin, and vancomycin individually, thus limiting the spectrum of microorganisms. Despite slight modifications in the development of the disease, none of them in isolation reduced the severity of uveitis as drastically as when they were used in combination. This could indicate that the bacterial origin of antigenic cross-reactivity is not limited to a single type of microorganism, but that several species of the microbiota contribute to uveitis [55].

Despite their obvious usefulness, broad-spectrum antibiotics also lead to an increase in resistant bacterial strains, which is a serious public health problem [41]. This makes this option not quite as interesting as it might at first seem and leads to the search for drugs with a narrower spectrum that eliminates targeted communities of bacteria, such as highly specific immunoglobulins. This approach has been suggested by Okai and colleagues as a potential treatment for IBD [74,75] using immunoglobulin A (IgA), an efficient modulator of the intestinal microbiota. These authors selected IgA monoclonal antibodies (clone W27) from the small intestine of healthy mice (which in vitro selectively bind to potentially pathogenic commensal bacteria such as *Escherichia coli*, but not to supposedly beneficial ones such as *Lactobacillus casei*); and found that its oral administration effectively prevented the development of the disease in experimental models of mice with colitis [75].

### 4.4. Faecal Microbiota Transplantation (FMT)

Another way to modulate the microbiota is the use of FMT, a procedure in which fecal matter from a healthy donor is transferred to a sick recipient. Its use is being investigated in a wide variety of diseases, and it has been approved for the treatment of recurrent colitis by *Clostridium difficile*, for which it has demonstrated its effectiveness [76]. FMT from patients with BD [22] or VKH [65] to EAU mouse models exacerbated the activity of their uveitis, with increased production of IL-17 and IFN-γ. However, due to its great interindividual variability, among other reasons, there is still not enough clinical evidence for its use in other diseases such as IBD, AS, and uveitis [77].

### 4.5. Immunomodulatory Drugs

The use of immunomodulatory drugs belonging to the group of “Disease-modifying anti-rheumatic drugs” (DMARDs) suppresses the growth of different bacteria, fungi, and viruses, and it is postulated that they could improve the severity of uveitis by acting through the intestinal microbiota [16,78]. Sulfasalazine, like other immunomodulators, has antibiotic functions and reduces vascular permeability, and improves both joint disease in patients with AS [79] and uveitis associated with HLA B27. On the other hand, dysbiosis of patients with active VKH decreased after immunosuppressive treatment with CsA, which also resolved intraocular inflammation, suggesting the effect of immunosuppressants on the microbiota [65].

Recently, an immunomodulatory effect of methotrexate and mycophenolate mofetil potentially linked to changes in the intestinal bacterial composition [80], which is specific and distinct for each drug, used in the EAU murine model has been suggested. Specifically, methotrexate at low maintained doses was able to decrease the adaptive, effector, and regulatory cellular response, both in the eye and in other tissues. This immunomodulatory effect correlated with specific changes in the composition of the gut microbiome. In contrast, mycophenolate induced an increase in highly suppressive Treg lymphocytes, showing a less suppressive effect on effector T populations in the eye and other tissues. This immunomodulatory effect was also proportional to differences in intestinal microbial composition after treatment [80].

### 4.6. Biological Drugs

Biological drugs have the ability to modify the natural course of numerous inflammatory diseases. A better understanding of the role of cytokines has led to the publication of studies supporting the use of anti-cytokine drugs (anti-TNF and anti-IL-6) in NIUs [33]. Adalimumab (ADA, anti-TNF drug) has been successfully used in the management of uveitis in patients with AS [81] and VISUAL I, II, and III clinical trials [82,83,84] have demonstrated efficacy and safety in non-anterior non-infectious, corticosteroid-refractory uveitis or in which corticosteroids cannot be used at acceptable doses. It is the only biological drug approved for this indication in the US and Europe [82,85]. In addition, Infliximab (IFX, anti-TNF drug) is approved in Japan for BD-associated uveitis, where it has demonstrated good tolerance and efficacy [86]. Nonetheless, this is not the case in other countries, where it must be used off-label.

Previous studies have suggested that treatment with anti-TNF restores the composition of the gut microbiota in intestinal [87] and extraintestinal [23] autoimmune pathologies. These are described in Table 3. Furthermore, there is increasing scientific evidence suggesting that the gut microbiome could be an indicator of clinical response to anti-TNF treatment and could play an important role in the efficacy of the drug [88]. Ribaldone et al. [87] evaluated in patients with Crohn’s disease (CD) the modification of the microbiota at baseline and six months of ADA treatment and found a decrease of *Proteobacteria*, which abundance is associated with dysbiosis and a diseased state, and an increase of *Lachnospiraceae*, composed mainly of anti-inflammatory butyrogenic species, in responder patients. This is in line with the idea that anti-TNF therapy restores the intestinal “eubiosis”, i.e., the balance of the intestinal microbial ecosystem. Similarly, Zhou et al. [89] also observed a restoration of the gut microbiota diversity in patients with CD that responded to IFX. In these patients, the abundance of *Clostridiales* increased to the levels detected in healthy individuals, but not in non-responder patients. In addition, predictive patterns of good response to IFX treatment have been identified [89]. Restoration of the gut microbiota has been also reported in patients with AS treated with ADA [23]. In this study, the differences in the intestinal microbiota of patients with AS were restored after 6 months of treatment with ADA, reaching a state similar to healthy controls by restoring the normal proportion of *Bacteroidetes* and *Firmicutes*, without finding differences between responders and non-responders [23]. If this association between the gut microbiome and clinical response to anti-TNF is confirmed, it could be used as an indicator of response to treatment before the start of therapy, increasing the accuracy of its indication. However, there are no published studies in patients with uveitis to date regarding the effect of anti-TNF therapy on microbiome status.

The above-mentioned studies have provided compelling evidence for the involvement of the microbiome in the pathogenesis and progression of NIU and have demonstrated the potentially beneficial effects of several treatment approaches that have an effect, directly or indirectly, on the microbiome, such as supplementation of probiotics or prebiotics, FMT, DMARDs and biological drugs. Probiotics and prebiotics, through direct modulation of the microbiome, have demonstrated an enhancement of the anti-inflammatory response of the intestinal immune system in animal models by increasing gut antimicrobial peptide expression [56] and decreasing the production of pro-inflammatory cytokines in the retinas [44] and cervical lymph nodes [57]. In addition, supplementation of prebiotics, mainly SCFAs, modifies the balance of T cells, switching from pathogenic Th17 to Treg [44] and decreasing induction of effector T cells [42]. In patients with BD, a reduced butyrate production by their microbiota has been linked to an increased inflammatory status [22,63], and a clinical trial is currently underway to determine whether butyrate supplementation could improve clinical manifestations of these patients by modulating their gut microbiota [63]. Unfortunately, literature on the effect of probiotic/prebiotic supplementation on the inflammatory level of patients with NIU is lacking, but evidence derived from animal models suggests that it may be a treatment option to attenuate the severity of uveitis [42,44,56,57]. FMT may also be considered a promising therapeutic approach in NIU. It has been demonstrated that FMT from patients with autoimmune diseases associated with uveitis, such as BD [22] and VKH [65], exacerbate the activity of the uveitis in EAU mice. Therefore, it is reasonable to hypothesize that FMT from healthy donors could directly improve the inflammatory status of NIU by introducing beneficial species into the microbiome. Regarding DMARDs, one study in VKH patients showed partial restoration of their microbiota to a state more similar to that of healthy controls after treatment and identified microbial markers predictive of treatment response [65]. Therefore, DMARDs treatment of NIU could also induce microbiota recovery, at least partially, thus decreasing disease severity. Finally, biologic drugs have proven their effect on microbiome modulation in immune-mediated diseases such as CD, IBD, and AS [23,87,89]. Recent evidence on ADA and IFX treatment of patients with these immune-mediated diseases shows a trend towards microbial restitution, which was more evident in those patients who responded to therapy [87,89], but with differentially altered bacteria in each study. A remarkable finding of these studies was the different microbial patterns observed in responders compared to non-responders to the biological therapy, which allowed the identification of microbial predictive markers of response to treatment. Although these results are promising in the area of personalized medicine, no such studies have been conducted on patients with NIU. If the association between the gut microbiome and clinical response to anti-TNF observed in other immune-mediated diseases is confirmed in NIU, the study of the gut microbiome would emerge as a useful tool for the implementation of personalized medicine in NIU.

## 5. Conclusions

We currently have growing scientific evidence supporting the causality between intestinal dysbiosis and disease induction by altering the host’s homeostatic immune status, with speculation of a possible association between an altered microbiome and the pathogenesis of intraocular inflammation. However, there are still some unknowns in the relationship between the microbiome and uveitis, as microbial mono-association studies that demonstrate causality of changes in the microbiome with the development or maintenance of uveitis are very rare. We review the most recent studies associating intestinal dysbiosis and uveitis, both in animal and human models. The ability to modulate the composition of the microbiota through dietary supplementation or the use of drugs such as antibiotics, immunomodulators, or biologics opens a new therapeutic line for this disease. In addition, although new studies are needed to confirm this, an association between the intestinal microbiota and the clinical response to anti-TNF seems likely, which would allow personalizing and monitoring of the treatment of these patients more accurately.

We believe that this review will be of great use to research groups working in this field since it compiles all the information published to date.

## Figures and Tables

**Table 1:** Pre-clinical studies with experimental autoimmune uveitis (EAU).

Authors	Study Type	Modulation–Intervention		Findings
**Horai et al., 2015** [35]	Experimental Spontaneous EAU	Decreased bacterial load of the microbiota	Combination of antibiotics (ampicillin, metronidazole, neomycin, and vancomycin) Germ-free mice	Decrease in the severity of the disease Decreased IRBP-specific T cells in the lamina propria of the intestine
**Zárate-Baldés et al., 2017** [55]	Experimental Spontaneous EAU		Antibiotics [35] separately: (ampicillin, metronidazole, neomycin, and vancomycin)	Slight modifications of the disease, but do not decrease the severity of uveitis Do not decrease the IRBP-specific T cells in the lamina propria of the intestine
**Nakamura et al., 2016** [41]	Experimental Induced EAU		Oral or intraperitoneal antibiotics (metronidazole, vancomycin, neomycin, ampicillin) Start one week before induction Individually and in combination	Decreased severity of uveitis in mice treated with oral antibiotics, but not intraperitoneal Separate oral metronidazole and vancomycin also decrease inflammation, but not neomycin or ampicillin Ampicillin, metronidazole, and vancomycin increase antibiotic-resistant bacteria of the class *Gammaproteobacteria* and the family *Enterobacteriaceae* Treg induction in intestinal lamina propria at week 1, and in extraintestinal lymphoid tissues and the eye subsequently Decreased effector T and inflammatory cytokines in cervical and mesenteric lymph nodes at week 1
**Heissigerova et al., 2016** [54]	Experimental Induced EAU		Germ-free mice Treatment with oral antibiotics (metronidazole and ciprofloxacin) Start one week before or on the day of induction	Decreased severity of uveitis in germ-free mice and mice treated with the antibiotic cocktail from one week before induction In germ-free mice: Reduced infiltration of macrophages and T cells into the retina, lower levels of IFN-γ- and IL-17-producing T cells, and higher levels of regulatory T cells into the drainage lymph nodes of the eye
**Dusek et al., 2020** [56]	Experimental Induced EAU	Supplementation with probiotics	2 live oral probiotic bacteria EcN and EcO 4 treatment regimes	EcN: protects against EAU EcO: non-protective Treatment with EcN is only effective if given prophylactically The protective effect is accompanied by the strengthening of the integrity of the intestinal mucosa, enhancing the anti-inflammatory function of the immune system of the intestinal mucosa
**Kim et al., 2017** [57]	Experimental Induced EAU		Antibiotics + probiotics IRT-5 (Lactobacillus casei, Lactobacillus acidophilus, Lactobacillus reuteri, Bifidobacterium bifidum y Streptococcus thermophilus)	Reduce the severity of uveitis after 3 weeks of IRT-5 Decrease effector T cells (CD8) and the concentration of pro-inflammatory cytokines in cervical lymph nodes The proportion of Treg in cervical lymph nodes was significantly lower in mice treated with IRT-5, suggesting that in this EAU model the modulation of effector T by IRT-5 is not mediated by Treg
**Chen et al., 2017** [44]	Experimental EAU	Supplementation with prebiotics	Sodium butyrate (NaB)	Attenuated ocular inflammatory response at 14 days after immunization Decreased inflammatory cell infiltration and inflammatory cytokine production in the retinas Decreased the frequency and number of Th17 and increased the frequency and number of Treg in both draining lymph nodes and spleen
**Nakamura et al., 2017** [42]	Experimental Induced EAU		Oral propionate	Attenuates uveitis Reduces the transport of effector cs T between the intestine and the spleen Decreases induction of effector T in cervical and mesenteric lymph nodes Increases Treg in intestinal lamina propria and cervical lymph nodes They demonstrate, for the first time, the increase in the traffic of leukocytes from the gastrointestinal tract to the eye in the EAU

IRBP: inter-photoreceptor retinoid-binding protein. EcN: *Escherichia coli Nissle 1917*. EcO: *Escherichia coli O83:K24:H31.*

**Table 2:** Clinical studies in which the causality of NIU has been related to the microbiota, and different therapeutic strategies for its modulation.

Authors	Disease	*n* Patients and *n* Control	Findings
**Kalyana et al., 2018** [60]	NIU	13 NIU vs. 13 healthy	Decrease in the abundance and diversity of gut bacterial microbiota in NIU Decreased diversity of potentially anti-inflammatory butyrate-producing bacteria in NIUs (*Lachnospira, Ruminococcus, Bacteroides, Dialister, Clostridium, Faecalibacterium, Roseburia, and* members of the Families *Lachnospiraceae and Ruminococcaceae)* Increased bacteria described as pro-inflammatory in NIU (*Prevotella copri)* Increase of potentially pathogenic bacteria in NIU (*Streptococcus)* Decreased anti-inflammatory probiotic potentials in NIU (*Bifidobacterium adolescentis and Bifidobacterium longum)*
**Jayasudha et al., 2019** [61]		14 NIU vs. 24 healthy	Decreased fungiome diversity in NIU Increase in opportunistic fungal species in NIU: *Malassezia restricta, Candida albicans, Candida glabrata, Aspergillus gracilis* Decrease of species with anti-inflammatory or anti-pathogenic properties: yeasts, (24 genera)
**Huang et al., 2018** [62]	AAU	38 AAU vs. 40 healthy	Increase of 7 fecal metabolites in AAU (6-deoxy-D-glucose 1, linoleic acid, N-acetyl-beta-D-mannosamine 3, shikimic acid, azelaic acid, isomaltose 1, and palmitoleic acid) No differences in gut microbiota between AAU and healthy subjects
**Consolandi et al., 2015** [63]	BD	22 BD vs. 16 healthy	Decrease in butyrate producers: *Roseburia* and *Subdoligranulum* in BD Reduction of butyrate production and increase of acetate in BD
**Yasar et al., 2020** [21]		27 BD vs. 10 healthy 3 clinical forms of BD	More abundant in BD and healthy: Prevotella, Faecalibacterium, Bacteroides, Blautia, Bifidobacteria Relative increase in Actinomyces, Libanicoccus, Collinsella, Eggerthella, Enetrohabdus, Catenibacterium, and Enterobacter in BD Reduction of Bacteroides, Cricetibacter, Alistipes, Lachnospira, Dielma, Akkermansia, Sutterella, Anaerofilum, Ruminococcease-UCG007, Acetanaerobacterium, and Copropaacter in BD In the three clinical forms of BD: Prevotella and Faecalibacterium were the most abundant Ocular BD (uveitis): increased Lachnospiraceae NK4A136. Presence of 2.5% Traponema (absent in the other 2) BD mucocutaneous: increased Dialister, Intestinomonas, and Marvinbryantia BD vascular: increased Gemella
**Shimizu et al., 2016** [64]		12 BD vs. 12 healthy	Phylum: increased Actinobacteria (and Lactobacillus), including Bifidobacterium; decreased Firmicutes, especially Clostridia in BD Generates: increase in Bifidobacterium and Eggerthella, decrease in Megamonas and Prevotella in BD
**Ye et al., 2018** [22]		32 BD vs. 74 healthy	Increased sulfate-reducing bacteria (*Bilophila* spp.) and opportunistic bacteria (*Parabacteroides* spp. and *Paraprevotella* spp.) in BD Decrease in butyrate-producing bacteria (*Clostridium* spp.) and methanogenic bacteria (*Methanoculleus* spp., *Methanomethylophilus* spp.) in BD Modulation: FMT from BD patients to experimental mice exacerbated the activity of their EAU
**Ye et al., 2020** [65]	VKH	82 VKH vs. 63 healthy	Increase in Gram-negative bacteria in VKH Decrease in butyrate, lactate, and methanogen-producing bacteria in VKH HLA-DRA (VKH susceptibility) was correlated with the presence of *Bacteroides sp.2.1.33B* and *Paraprevotella* free, and the absence of *Alistipes finegoldii* and *Eubacterium eligens* Modulation: partial restoration of the microbiota in VKH after immunosuppressive treatment with corticosteroids and CsA. They identify species associated with good response to treatment Modulation: FMT from VKH patients to experimental mice exacerbated the activity of their EAU

EAU: Experimental autoimmune uveitis. NIU: Non-infectious uveitis. VKH: Vogt-Koyanagi-Harada syndrome. AAU: Acute anterior uveitis. BD: Behçet disease. FMT: Faecal microbiota transplantation. CsA: Cyclosporine A.

**Table 3:** Clinical studies in other autoimmune diseases (not uveitis).

Authors	Disease	*n* Patients and *n* Control	Study Type	Findings
**Tito et al., 2017** [68]	AS	27 AS vs. 15 healthy	Causality	Correlation of histological intestinal inflammatory status with microbiota profile in AS Increased Bacterial Species *Dialister* correlated with inflammatory activity (Ankylosing Spondylitis Disease Activity Score) → *Dialister* May be a marker of AS activity
**Costello et al., 2015** [67]		9 AS vs. 9 healthy		Correlation between terminal ileum microbiota composition and disease status (AS) Increased abundance of five families of bacteria in AS: *Lachnospiraceae, Ruminococcaceae, Rikenellaceae, Porphyromonadaceae,* and *Bacteroidaceae* Decrease of two families in AS: *Veillonellaceae* and *Prevotellaceae*
**Chen et al., 2021** [23]		30 AS vs. 24 healthy	Modulation	Decreased microbiota diversity in AS ADA treatment (6 months): Restoration of the intestinal microbiota in AS No statistically significant differences between responders and non-responders, but a greater abundance of *Comamonas* was found in non-responders Depletion of *Bacteroides* and *Megamonas* and enrichment of *Collinsella* Decreased *Dialister* that is restored with treatment (unlike [66])
**Ribaldone et al., 2019** [87]	IBD	20 CD pre- and post-ADA treatment		Treatment with ADA (6 months): trend towards the restitution of intestinal “eubiosis” in responders
**Zhou et al., 2018** [89]		72 CD, 51 UC vs. 73 healthy 16 CD pre- and post-IFX		Relative increase in *Actinobacteria* and *Proteobacteria (Enterobacteriaceae)* and decrease in *Firmicutes (Clostridiales)* were associated with the severity of IBD Treatment with IFX (30 weeks): Restitution of intestinal microbiota diversity and relative increase in *Clostridiales* in patients responding to the drug

AS: Ankylosing spondylitis. IBD: Inflammatory bowel disease. ADA Adalimumab. IFX Infliximab. CD Crohn’s Disease. UC Ulcerative colitis.
